# Telework and Lifelong Learning

**DOI:** 10.3389/fsoc.2021.642277

**Published:** 2021-03-29

**Authors:** Cecilia Bjursell, Ingela Bergmo-Prvulovic, Joel Hedegaard

**Affiliations:** Encell–National Centre for Lifelong Learning, School of Education and Communication, Jönköping University, Jönköping, Sweden

**Keywords:** lifelong learning, sustainable working life, social inequality, telework, telecommuting

## Abstract

The increase of telework during the pandemic is predicted to impact working life, not only in terms of a larger number of employees working from home, but more importantly, it may transform the way we conceptualise work. This will in turn impact systems for and participation in lifelong learning. There is a risk for increased social inequalities, as neither telework nor lifelong learning is evenly distributed among workers. Statistics on telework in the EU show that there are differences between age groups, nations, sectors, and professions. If these trends will steer forward, there is a risk of widening gaps between countries, companies, and workers. To establish the current knowledge base, we have gathered literature reviews from several disciplines. One finding is that the previous literature on telework has not included lifelong learning in any form (formal, non-formal and informal). Based on a review of previous studies, we suggest a number of research questions for future research. This is relevant as research about telework and lifelong learning has the potential to contribute to a sustainable working life in terms of providing more flexible arrangements for employees and to support the lifelong learning that takes place in contexts such as the office, home, online meetings, and virtual reality.

## Introduction: Increased Telework and Increased Social Inequalities

In 2020, telework has become the new normal in working life. Considering employees in the EU between the ages of 15 and 64, an average of 5.4% worked from home in 2019 (Eurostat, 2020). These numbers have been similar for 10 years. However, the number of people who work from home a few days a week has increased during the same period, from 5.2% in 2009 to 9% in 2019. Among the self-employed, almost one in five worked from home. There are differences between men and women and between different ages. Slightly more women than men worked from home (5.7 vs. 5.2% in 2019). Moreover, the proportion of people who work from home increases with increasing age. Among people in the 15–24 age group, 2.1% worked from home, while among people in the 50–64 age group, 6.6% worked from home. (Eurostat, 2020).

However, all these figures are from the time before the pandemic, and the proportion of people working from home has increased disproportionately. In Sweden, it increased tenfold, and among white collar workers, two thirds have worked from home since the pandemic struck in 2020 (Internetstiftelsen, 2020). Most people who have been working from home are satisfied, while those who have studied at a distance are dissatisfied (ibid). Although working from home may not remain to the same extent as the pandemic subsides, it is likely that we will see an increase compared to the figures from 2019. Not least, the proportion who will combine work remotely with presence in the workplace may increase. However, the ability to choose and combine workplaces is not evenly distributed between professions and positions. The rapid changes in working life also raise the question of competence. In Sweden, a land often ranked as highly digitalised, a survey showed that during the last year, 49% have felt that they have insufficient digital knowledge both in the labour market and privately (SVT, 2021). In the World Economic Forum’s “Future of jobs report 2020”, it is estimated that around 40% of workers will require reskilling of six months or fewer, and a staggering 94% of business leaders expect employees to pick up new skills on the job, compared to 65% in 2018. The needed skills include analytical skills as well as skills in self-management, such as active learning, resilience, stress tolerance and flexibility (WEF, 2020). Self-management skills are innately connected to telework, as it is a situation that requires more responsibility from the employee in terms of managing the physical and psychological work aspects, as well as performing the work itself. To support a sustainable working life, in light of this year’s changes linked to telework, the following insights presented by the European Commission (2020) can guide efforts:

• There are large differences in the prevalence of teleworking between EU Member States, between sectors and between professions.
• The readiness to implement teleworking on a large scale is high in IT and knowledge-intensive sectors and for the highly educated, but there are large differences between countries.
• In many EU countries, more than half of those who now work remotely had not tried it before.
• If previous trends will steer forward, the risks of widening gaps between countries, companies, and workers are great.

Although most people expect that they will return to the workplace after the pandemic, it is highly probable that the number of people teleworking will increase to some extent, and this will change the conditions for working life in several ways. Not only will it change the social dynamics of workplaces, but we also need to discuss the dissatisfaction among people in distance studies in connection to competence development and upskilling. There is a risk that efforts of improvement, such as the provision of flexibility through telework or lifelong learning through education and training, will be unevenly distributed among professions and sectors. There is, because of this, a risk for increased social gaps in society. When it comes to the relationship between teleworking and lifelong learning, there are at least two ways to approach this connection. Firstly, one can focus on the individual’s perspective and how teleworking affects formal, informal and non-formal lifelong learning. Secondly, the focus can be on lifelong learning as policy, where an important message is that everyone should have the opportunity to participate in society on equal terms.

Based on the assumption that telework will increase compared to the frequency of teleworkers before the pandemic, and the fact that there is an urgent need for new skills, the aim of the paper is to review previous research on telework. The review is guided by the research question: What can be concluded about telework based on previous research, with regard to efforts for competence development and upskilling as part of the individual’s lifelong learning? In this paper, we will make a synthesis of reviews addressing telework to find out what we already know about the impact on lifelong learning, including competence development. Based on this, the discussion addresses possible consequences that teleworking might have for an individual’s lifelong learning and proposes questions for further research. The paper is based on a multidisciplinary and broad understanding of telework, as suggested by Allen et al. (2015), to fully understand the benefits and drawbacks of working from home.

## Telework: An Overview

In 1973, Jack Nilles’ book *The Telecommunications-Transportation Tradeoff* introduced the term telecommuting to discuss distance working as a solution to traffic congestion and pollution. A decade later, companies saw telework as a tool to reduce the expense for office space, but this has shifted again into telework as a strategy to attract and retain top personnel (Kurland and Bailey, 1999). During the pandemic, employees have called attention to the risk that employers (again) might see telework as a tool to reduce expenses for office space. However, what has been raised in research, as discussed below, is that telework might require investments from the employer to achieve the desired benefits. Therefore, the cost structure might be different, but it should not necessarily be understood as primarily a cost-cutting strategy. Kurland and Bailey (1999) defined four types of telework:

• *Home-based telecommuting* refers to when employees on a regular basis work from home, but they are based at a central office belonging to an employer.
• *Satellite offices* refer to work locations situated at a convenient location allowing employees to cut the time they spend commuting. This branch office is furnished and equipped by the employer.
• *A neighbourhood work centre* is like a satellite office, with the exception that several employers share the lease of the building and it may have a site owner responsible for the location.
• *Mobile workers* are employees who work in an assortment of locales, such as from home, from a car, from a plane, or from a hotel.

Telework can thus take place in different contexts, but when telework is researched, the focus is usually on home-based telecommuting. According to the four types of telework presented by Kurland and Bailey (1999), telecommuting is a form of telework; in later papers, however, there is often no separation between telecommuting and telework when addressing working from home. The concepts telecommuting and telework are used interchangeably in this paper.

When looking at who participates in telework, why they do it and what happens when they do, Bailey and Kurland (2002) find that telework is a complex concept and phenomenon, although in research, the focus is often on one or a few parameters. In their review, they established that the individual teleworker was a nearly universal focus of study and that there were assumptions that telework took place on a full-time basis. Furthermore, Bailey and Kurland highlighted that, apart from methodological weaknesses, the studies lacked awareness of issues of status and power. Although the demographics of teleworkers are elusive, the teleworking population may be divided along occupational and gender lines. Later reviews and frameworks for studies on telework list similar advantages and challenges of telework (Crandall and Gao, 2005; Pyöriä, 2011; Boell et al., 2013). In Tables 1, 2, lists of advantages and challenges from reviews of telework are presented. The collection is not complete; for example, the list in Crandall and Gao (2005) is based on Baruch (2001) and Daniels et al. (2001). This description of previous research will provide a background to the discussion on lifelong learning and telework.

**TABLE 1:** Advantages of telework on the individual and the organizational level.

Advantages with home-based telework	References
Individual advantages: less time consuming, cost savings, less stress, no need for relocation, more autonomy, schedule flexibility, comfortable work environment, fewer distractions, absence of office politics, work/family balance, workplace fairness, and more job satisfaction.	Kurland and Bailey (1999)
Organizational advantages: greater productivity, lower absenteeism, better morale, greater openness, fewer interruptions at office, reduced overhead, wider talent pool, lower turnover, and regulation compliance (e.g., disabilities act).	
Individual: Higher job satisfaction, higher organizational commitment, less pressure, better time management, reduced travel time, balanced work and home life, distraction-free environment, less involvement in office politics, suitable for homebound employees.	Crandall and Gao (2005)
Organizational: increase productivity, lower costs, less office space needed, reduced absenteeism, lower turnover, do not have to have all employees in one location (a terrorist consideration), increased recruitment options, and able to adapt to the virtual organization.	
Telework is environmentally friendly, telework can create more flexible work arrangements and at the same time help to lower the costs of running office premises, telework can be a way of raising the company’s corporate image, changeover to telework has improved job control and well-being at the individual level and increased the overall efficiency of organizations.	Pyöriä (2011)
Individual: Savings based on less travelling and type of clothing, possibility to coordinate for work-life balance, spatial mobility beyond commuting distance, increased work autonomy, increased job satisfaction, and increased productivity.	Boell et al. (2013)
Organizational: Recruitment and retention, increased work morale, productivity gains, improved agility, and financial advantages such as cost savings for office rent.	
Increased perceptions of autonomy and lower work–family conflict; good quality of employee–supervisor relationship; job satisfaction; lower turnover intent and role stress; higher supervisor ratings. These beneficial consequences appeared to be at least partially mediated by perceived autonomy.	Gajendran and Harrison (2007)

**TABLE 2:** Challenges of telework on the individual and the organizational level.

Challenges with home-based telework	References
Individual challenges: Social isolation, professional isolation, organizational culture, reduced office influence, work/family balance, informal interaction, conducive home environment, focusing on work, longer hours, access to resources, and technological competence.	Kurland and Bailey (1999)
Organizational challenges: Performance monitoring, performance measurement, managerial control, mentoring, jealous colleagues, synergy, informal interaction, organization culture, virtual culture, organization loyalty, interpersonal skills, availability, schedule maintenance, work coordination, internal customers, communication, guidelines (e.g., expenses), and technology.	
Individual: Feelings of isolation from the work culture, lack of promotional opportunities, loss on the assignment of good projects, dissatisfaction with peer relationships, less influence over the people and events at work, work/family conflict, and harder to take a sick day.	Crandall and Gao (2005)
Organizational: More difficult to supervise, assessment concerns, special logistics requirements, sensitive information could be compromised, goes against the concept of teamwork, control over health and safety, and lack of infrastructure support (secretary, etc.).	
The importance of agreeing on a framework for telework, telework does not suit everyone, the problem of a traditional management culture, teamwork, and data security.	Pyöriä (2011)
Individual: Work-life blurring, lack of socializing opportunities, questions about career, less workplace involvement, reduced trust, lack of technical support, and unwanted interruptions.	Boell et al. (2013)
Organizational: Management practices do not fit the situation, legal framework is not sufficient, hinders teamwork and collaboration, lack of relevant expertise and training, infrastructure, and technology outside the office, data security, and investments in telework costs.	
High-intensity telecommuting (<2.5 days a week) harmed relationships with co-workers.	Gajendran and Harrison (2007)

The advantages for the individual are that telework provides flexibility and autonomy. High autonomy is a factor supporting evidence for home-based telecommuting as an employee-oriented human resource practice (Hornung and Glaser, 2009). Workers who can use telework to adjust tasks to meet their needs and desires are more likely to be satisfied, and telework is more positively related to firm performance than other dimensions of labour flexibility (Martínez-Sánchez, et al., 2008). Less time spent commuting provides more time for other activities. The individual has more job control and opportunities to manage the work-family balance. To be in control could be one explanation for other positive outcomes, such as less stress, job satisfaction, and well-being. Fewer interruptions when working and cost savings are also mentioned as advantages. An empirical study of 102 employees from a large United States government agency reported that employees experienced more job-related positive affective well-being when teleworking compared to when working in the office, but the individual differences moderated this relationship (Anderson et al., 2015). The discussion focuses on the need to consider the affective consequences of telework and the characteristics that determine who will benefit from working at home. Telework before the pandemic was in some cases regarded as a reward, a perspective that could explain positive notions of telework (Gajendran and Harrison, 2007).

The organizational advantages are increased productivity and increased work morale. That telework is perceived to improve performance, increase productivity, and strengthen organizational commitment was supported in an analysis of empirical studies on telework and organizational outcomes (Harker Martin and MacDonnell, 2012). Fewer interruptions and cost savings are also mentioned as advantages for the employer. Access to a wider talent pool creates advantages for recruitment, and retention of personnel provides stability in operations. Regulations compliance is supported, and the Disabilities Act is mentioned as an example of this. Telework is sometimes presented as a way for employers to work with inclusion and diversity perspectives, but this is an area where more research is needed. In a literature review with a focus on work-life balance for workers with disabilities or workers who have family members with disabilities, the authors found a total of only 48 articles over 20 years (Igeltjørn and Habib, 2020). The review indicates that although several articles imply that policies could have positive effects on the work environment of home-based teleworkers, they do not describe how this could be achieved or whether existing policies have yielded the desired effects.

Telework also presents challenges, listed in Table 2. In reading these challenges, the reader should be reminded of the meta-analysis of psychological mediators and individual consequences performed by Gajendran and Harrison (2007) that provided evidence against the telecommuting paradox in variables listed as advantages AND challenges (for example, family-work conflict). There are thus contradictions between the studies referenced in this paper, and we want to raise awareness of the methods used and how they impact what is studied, how findings are presented, and the relevance of results in relation to practical situations. Therefore, continued analyses and various ways to study the details of telework are still needed, not the least during and after the pandemic situation.

The challenges for the individual when teleworking can be social and professional isolation, with less influence and involvement in matters that are dealt with at the office. The negative emotional impact of teleworking can come in the form of emotions such as worry, irritability, guilt, and loneliness (Mann et al., 2000; Mann and Holdsworth, 2003). Detachment from work is described as a career risk, as the individual might miss out on opportunities when not being at the office in person. Limited access to organizational resources and ergonomic issues in the home environment are other drawbacks. Work–family balance was an advantage, but it can also be a challenge, depending on the individual’s situation. The challenges for the organization concern management activities, such as monitoring, measuring, and controlling employees’ outcomes. This “problem” should, however, be viewed in the light of findings stating that telecommuting availability was directly and indirectly related to engagement via perceived supervisor goal support, and that the option to telecommute could increase employee engagement (Masuda et al., 2017). Control over health and safety, including data security, are challenges when employees work from home. Pain or discomfort stemming from telework could be addressed with teleworker ergonomics training. A study found that almost half of the participants experienced physical problems, but 85% of the participants had not received ergonomics training (Harrington and Walker, 2004). A challenge for both the organization and the individual is lack of informal interaction, which has consequences for the development of interpersonal skills and mentoring opportunities.

Crandall and Gao (2005) included an outlook on unresolved and emerging issues that are necessary to include for a complete understanding of telework conditions. The first unresolved issue concerns the role of the organization and government in establishing employee safety when working from home. In many countries, the employer has an obligation to maintain a safe working environment regardless of where the work activity is performed. When it comes to telework situations, questions about insurance coverage and responsibility may arise because of blurred boundaries. The second unresolved issue addresses whether telework is a way for the organization to exploit workers. Two main targets have been identified in this debate: women and less skilled workers. These connect back to previous research findings that it is relevant to include occupation and gender to understand differences in the teleworking population (Bailey and Kurland, 2002). Whether telework is a form of exploitation directed at women remains speculative; however, it is highly probable that telework can contribute to increased polarization in working life and that this polarization could be along gender lines (Crandall and Gao, 2005). The third issue that Crandall and Gao (2005) suggest for discussion is the use of technology, as this is a field in rapid development. According to the desktop metaphor, telework relies on a standard set of technology such as PC, e-mail, etc., but the virtual reality metaphor is emerging as an alternative. Virtual reality potentially offers increased social richness and a feeling of “being there” (that is, being at the office). Technology, and the emergent new society connected to technological development, enables telework in new ways. However, in the information society, it is increasingly difficult to define or demarcate working hours and places of work and to distinguish between commodity and service production (Pyöriä, 2011). Hybrid work set-ups call for new human resources and management practices with regard to how social space and territoriality play out (Sewell and Taskin, 2015).

From the organizational point of view, the individual as well as the task dimension are relevant to understanding how to plan telework. This requires considering professional differences as well as task differences. Writing a memo could fit telework, whereas activities requiring physical coordination between colleagues require office presence. The type of work done may provide clear or blurred boundaries between work life and private life, and therefore not all professions have the same potential for telework (Hislop and Axtell, 2007). Bailey and Kurland (2002) suggested that future research should expand the lens beyond individual teleworkers to include the practice that telework affects, to consider why people work away from the office and include the option that telework may be a way to cope with the demands of the modern workplace, and lastly, to emphasise theory-building and connect links to organizational theories.

The individual dimension of telework discussed above is on an overall level. Several authors pointed to the fact that individual differences may influence the perception and outcome of telework. Factors such as family structure, living space and technological equipment constitute the conditions for telework. Age and experience could be relevant to understanding telework and could be connected to the ability to structure work efforts at home. When workers value the status associated with telework, or if telework satisfies needs for achievement and stimulation, it may be easier for organizations to allow larger proportions of their workforce to telework, as these values can motivate teleworkers to perform their tasks in line with organizational goals (Peters et al., 2016). Pyöriä (2011) pointed out that telework is suitable for jobs that require peace and concentration and in jobs that require creative problem-solving skills, where the option to work flexibly according to need and inspiration is vital (Pyöriä, 2011). The idea of the creative problem-solving professional being flexible to live and work anywhere and anytime stands in contrast to the tendency for knowledge workers to concentrate in and around economic hubs. Additionally, we are reminded that the notion of what technology can do for us often takes on mythological dimensions:

“The idea of the empowered teleworker has become highly charged symbols, in some instances a clear myth, incorporating an overtly optimistic vision of the almost limitless possibilities that ICTs have to offer” (Pyöriä, 2011, p. 387).

To implement telework as a practice in working life should furthermore give attention to the proper recognition of interests from unions, as labour representatives. In Europe, trade unions have discussed, for example, the burden of costs when working from home, occupational safety, ergonomics, and work hour arrangements, as part of formal frameworks for telework. Kurland and Bailey (1999) indicated that one risk with telecommuting is that worker solidarity decreases when telecommuters are physically dispersed and less able to organize collectively, which may affect the work of unions. The decreased orientation towards other workers may also be a problem for teamwork and thereby for learning:

“Additionally, managers may find it difficult to create team synergy and to overcome the absence of informal, interactive learning—learning that takes place by the water cooler, over lunch, or in the hallways” (Kurland and Bailey, 1999, p. 59).

The problem with telework is that interaction and communication often take place as scheduled meetings, which does not support learning in the same way as the informal interaction in the office does. This spontaneous learning cannot be scheduled, but it is nevertheless an important part of the individuals’ development, sometimes called “in place career development”. A specific part of this learning is to master interpersonal skills that may be needed to interact, communicate, and cooperate with colleagues, customers, students, and others as part of working. With a substantial part of the workforce working for a considerable amount of time from home, this could change the nature of social intercourse in unknown ways. Taskin and Bridoux (2010) have highlighted that teleworking could endanger an organization’s knowledge base and competitive advantage as the knowledge transfer between teleworkers and non-teleworkers is threatened. Apart from mentioning the risk for knowledge drawbacks, lack of informal learning, and the need for ergonomics training for teleworkers, the issue of competence development and lifelong learning is absent in the literature on telework, despite the recognition of new ways to conceptualise work, which may demand learning and require new theoretical frameworks as well as practical knowledge for individuals, organizations, and societies.

## Telework During the Pandemic and Lifelong Learning

The lack of research on how telework affects lifelong learning, competence development, and upskilling is problematic, and in addition to this, we now have the current situation with a pandemic changing our everyday lives, including how and where we work. While telework used to be a tool to make employment more attractive, it has currently become a measure to stop infection. When telework is performed by people who do not want to work from home and may have limited experience doing so, this will most likely change our understanding of telework. Similar to how 9/11 changed airport security jobs across the globe, the pandemic will change jobs in ways that we have yet to understand (Li et al., 2020). Moreover, families are locked up together in their homes, raising issues of places for the whole family to work and study, and whether the internet connection can handle several virtual meetings taking place in the same home. As people were forced to work from home when the pandemic hit in 2020, they were at the same time forced to learn. This meant that a massive digital competence boost took place, which provided insights into the need for HR practitioners and managers in general to understand the needs of teleworkers and provide adequate support. As mentioned in the introduction of this paper, people also learned that they did not have sufficient digital skills to manage everyday tasks in life and work.

Furthermore, we have not seen the full effects of actions taken and the impact of changes on our understanding of telework and the transformation of employees into remote workers. So far, the premises have been similar for all, regardless of age, gender, and level of expertise, although there is a big difference between people working in professions that can be performed at a distance, as opposed to those who have to be at work to perform their tasks. Compared to the few veterans already working from home, the newbies, individuals with limited experience of working from home (Li et al., 2020), had to master virtual meetings, online software, e-learning tools, and more during a short period of time.

“The reality is that most newbies have been forced to learn fast how to stay at the top of the game and ensure that they have the knowledge to follow the veterans. They have to engage with online forums, amend work documents, undertake online meetings, share resources, and make the argument online. They simply try to figure out what they have to do and, in many cases, without any support” (Li et al., 2020, p. 201).

Even though some learning that took place came in the form of competence development courses that were quickly developed by the organizations or by learning institutions, the lion’s share of the learning that took place concerned informal learning driven by concrete needs for solving problems. To talk of learning instead of education is not without consequences because it emphasises the individual’s development instead of the institutional context within which it may take place. This also entails a shift from the subject content to the human aspects that are involved; we move from merely teaching a subject to considering a person’s development (Jarvis, 2007). This idea is based on two fundamental principles for lifelong learning:

• Learning must be understood as a complete whole and as an interaction between the individual and their surroundings. When lifelong learning is part of the public debate, much is spoken about what the individual must learn in terms of specific competencies. In such a case, according to theories about lifelong learning, there is a lack of dialogue about what it is that gets the individual to engage in learning. In contrast to policy initiatives that base this on external driving forces (for example, a demand for certain competencies within a particular industry), in life-long learning, the question that is asked is: What is it that creates real interest in learning in the individual?
• The individual’s identity process is central to learning. Theories about lifelong learning claim that this includes a new understanding of oneself. In short, learning entails a renegotiation of one’s identity. For example, for many adults, learning about the digital world (that we live in) entails a significant adaptation with respect to their identity. An example of this is when a teacher, who may be an expert in the classroom, is a mere beginner with respect to the digital world. To understand learning in practice, one should ask the following question: What effect does participation in learning have on the person’s perception of themselves?

These two points are theoretically grounded (Wenger, 1998; Illeris, 2004; Jarvis, 2004, Jarvis, 2007, Jarvis, 2012; Illeris, 2017), but they also provide practical advice with regards to arrangements for adult education initiatives. When the labour market calls for competence development and upskilling, most often, it does not consider the individuals who are affected by this; instead, focus is placed on the benefits that the employer hopes to reap from such efforts. To move forward, one might ask: What is it that creates true enthusiasm in people? What internal effects does learning have on people? Are there structural and mental spaces within the organization for the individual who undergoes change through education? These are important questions for the person who wishes to support lifelong learning, and they are important to set in relation to the telework context. Based on the foundation set out here by lifelong learning theories, the following section provides a set of questions and ideas to guide research that could contribute to theoretical as well as practical knowledge.

## Future Research on Telework and Lifelong Learning

The reviews summarised above do not specifically address competence development or lifelong learning. Kurland and Bailey (1999) stated that one challenge for managers was that telework led to a focus on outcome rather than the process. Telework often means that a manager cannot see when employees are struggling and step in with reliable and constructive performance feedback. The situation of the manager who works with formative feedback is similar to that of a teacher, and the balance between focus on output and process in competence development at work should be addressed in future studies. Hybrid forms of work arrangements may be connected to new power structures, and the roles and responsibilities of managers as well as employees may evolve. To understand complex entanglements and to approach new, visible, and not visible challenges and changes in telework, socio-material approaches provide opportunities for insights (Boell et al., 2013; Sewell and Taskin, 2015). Spatial mobility and temporal flexibility change the nature of work itself, work processes, and human engagement. The development of virtual realities (VR) is often presented as a way to achieve increased social richness (Crandall and Gao, 2005), but we do not yet know what the impact of virtual realities will be on learning in a telework setting. Suggested research questions are:

RQ1: How does changed interaction patterns between manager and employee impact formative feedback in the organization?
RQ2: How will hybrid forms of work arrangements interplay with competence development in organizations?
RQ3: What does informal, non-formal and formal learning mean in a telework context?
RQ4: How will telework change in the a) nature of work, b) work processes, and c) human engagement influence individuals’ lifelong learning?
RQ5: How can the social richness in virtual realities compensate for the relational disadvantages of telework?

In the telework literature, flexibility and autonomy of workers are often presented as challenges, as they stand opposed to the need for teamwork and collaboration in the organization (Boell et al., 2013). This contradiction between autonomy and coordination may, however, be a chimera based on the idea that teamwork needs to be led by a manager. Self-organized teamwork could work well in many professions and may even be more effective than top-down management in several cases. Contact between team members based on emergent problems in the work process could be considered a strong key to informal learning, as learning at work is often problem based. This is an area where we need future studies to validate this proposition. Context and interaction are two key concepts in lifelong learning theories, and the challenges with interaction in telework, combined with context entanglements, propose that we may need to study telework in innovative ways to obtain the full picture.

RQ6: How will the balance between autonomy and teamwork influence individuals’ learning and competence development?
RQ7: How do individuals engage in the self-organization of learning, and what kind of support do they need from the organization?
RQ8: How can problem-solving initiatives in the individual’s daily work be enriched by reflection, and how is this included in a relevant way?

When telework some decades ago became an option for many due to the development of technology, there were hopes that telework would help organizations decrease real estate costs, promote a healthy work–family balance, aid compliance with Disabilities Act, and reduce air pollution and traffic congestion (Bailey and Kurland, 2002). What we have observed through the literature review results is that the advantages presented have not been realised to the extent that was expected. The cost for telework is not simply reduced, but investments in the home office are needed to avoid negative effects on the employees’ health. As a form of flexible work arrangement, however, telework has a natural place in today’s working life. Rather than considering telework and telecommuting as an option to working at the office, it should be regarded as a practical flexible arrangement that can boost productivity without resulting in social exclusion or jeopardizing crucial interactions with colleagues (Pyöria, 2011) and that can be used differently depending on the task and on the individual. The individual’s expectations and possibilities can be a starting point when setting up plans, but the organization should have knowledge about what is possible and why they are doing it.

RQ9: How can telework support an inclusive approach to learning and competence development?
RQ10: What kind of investments are needed to improve skills for and through telework?
RQ11: How do organizations embrace individuals’ expectations and possibilities to support lifelong learning in telework?
RQ12: What kind of organizational strategies are emerging concerning telework, and how do they include learning and competence development?

Before ending this section, it might be appropriate to remember that early research on telework proposed that telework could be a way to reduce traffic congestion and pollution—the environmental gains when people work from home. The hopes of an improved environment are still relevant, and research during and after the pandemic can shed some light on whether telework has changed the condition of our natural environment. This paper has, however, highlighted the need to recognize telework as an environment for learning. This is also important, as we need knowledge in this area to be able to support a sustainable working life where people thrive and can make robust contributions to companies and society.

## Conclusion

There are increased needs for competence development and upskilling due to the digitalization of our everyday lives, but digitalization also enables telework solutions of various kinds and with new tools. While telework and lifelong learning are usually discussed in positive terms, we want to raise attention to the fact that participation in both telework and lifelong learning is not evenly distributed in the population. This has become obvious during the pandemic where, for example, professions with a critical societal function, such as employees in health and social care, have not been able to telework. The transition to, and appreciation of, telework is probably highest among educated white-collar workers who already may have had some experience of telework and who are interested in participating in further development. Differences between age groups, nations, sectors, and professions should be kept in mind when formulating telework and/or lifelong learning practices and policies. If not, there is a risk of increased social inequalities between countries and between individuals.

The review in this paper has shown a lack of research on lifelong learning in all its forms (formal, non-formal and informal) in telework. Research about telework and lifelong learning has the potential to contribute to a sustainable working life in terms of providing more flexible arrangements for employees and to support the lifelong learning that takes place in different contexts (office, home, virtual, etc.). As there are expectations of an increase in telework, full-time or in hybrid solutions, after the pandemic has subsided, it is relevant to increase the knowledge about lifelong learning practices in this area.

## Data Availability

The original contributions presented in the study are included in the article/Supplementary Material, further inquiries can be directed to the corresponding author.
